# Predictability modulates neurocognitive semantic processing of non-verbal narratives

**DOI:** 10.1038/s41598-020-66814-z

**Published:** 2020-06-25

**Authors:** Emily L. Coderre, Elizabeth O’Donnell, Emme O’Rourke, Neil Cohn

**Affiliations:** 1 Department of Communication Sciences and Disorders, University of Vermont, Burlington, VT United States; 2 Department of Communication and Cognition, Tilburg School of Humanities and Digital Sciences, Tilburg Center for Cognition and Communication (TiCC), Tilburg University, Tilburg, The Netherlands

**Keywords:** Language, Reading

## Abstract

Predictability is known to modulate semantic processing in language, but it is unclear to what extent this applies for other modalities. Here we ask whether similar cognitive processes are at play in predicting upcoming events in a non-verbal visual narrative. Typically developing adults viewed comics sequences in which a target panel was highly predictable (“high cloze”), less predictable (“low cloze”), or incongruent with the preceding narrative context (“anomalous”) during EEG recording. High and low predictable sequences were determined by a pretest where participants assessed “what happened next?”, resulting in cloze probability scores for sequence outcomes comparable to those used to measure predictability in sentence processing. Through both factorial and correlational analyses, we show a significant modulation of neural responses by cloze such that N400 effects are diminished as a target panel in a comic sequence becomes more predictable. Predictability thus appears to play a similar role in non-verbal comprehension of sequential images as in language comprehension, providing further evidence for the domain generality of semantic processing in the brain.

## Introduction

Prediction in language processing remains a widely debated topic^1–6^, but it is less clear to what degree such predictability extends to other modalities. In studies of the brain using event-related potentials (ERPs), semantic processing is indexed by a negativity peaking around 400 ms after the presentation of a stimulus, the N400^7^. While there is still debate and discussion around the specific functions of semantic processing that this component reflects, such as semantic integration or retrieval of lexical information^8–10^ (see Kutas & Federmeier^7^ for a broader discussion), this component has long been shown to be sensitive to predictability^11^, making it central to research questions about prediction in language processing. Studies of predictability with the N400 have typically manipulated cloze probability^1,11,12^: the expectancy of a specific target word given the contextual constraints of a preceding sentence. Cloze probability is generally quantified by obtaining cloze ratings: measures of agreement about an upcoming target word in a sentence. In “high-cloze” sentences, a target word is highly predictable given the preceding sentence context (e.g. “He mailed the letter without a STAMP”), while in “low-cloze” sentences, it is less predictable (e.g. “The bill was due at the end of the HOUR”)^11^. Cloze ratings range from 0 (meaning that 0% of people would predict the same upcoming word) to 1 (meaning that 100% of people would agree upon a predicted word). High-cloze words generate a reduced N400 compared to low-cloze words, suggesting that the greater the predictability of a word given the constraints of the preceding sentence context, the smaller the N400 amplitude. This effect is continuous, such that cloze rating is inversely correlated with N400 negative amplitude (with larger negative amplitudes occurring for lower-cloze words)^12^.

Linguistic debates about the nature of predictive processing related to the N400 remain focused on the verbal modality. However, N400s have long been acknowledged as a default neural mechanism for semantic processing *across* modalities, as N400s have also been observed across modalities of pictures, sounds, and other sensory stimuli^7^. N400 effects to visual stimuli tend to be more anteriorly distributed than those found to linguistic stimuli^13–16^ and are also often preceded by a slightly earlier component called the N300, an anterior negativity peaking approximately 200–300 ms after stimulus presentation which is also modulated by semantic incongruencies^17–19^. The N300 has been associated with object categorization or identification^14,15^. While some studies have concluded that the N300 and N400 components reflect similar neurocognitive mechanisms^20,21^, others have concluded that the two are separable^14,15,22^.

Discourse and narrative research has accumulated a wealth of evidence suggesting that visual and linguistic narratives rely on domain-general cognitive mechanisms. For instance, as in sentences, incongruous or unexpected panels in a visual narrative sequence have been shown to evoke larger N400s than congruous panels^22–24^. Given these similarities between visual and linguistic semantic processing, and the wealth of research examining predictability in the linguistic domain, it is curious that only a few studies to date have discussed the role of prediction in comprehending visual sequences^23,25–28^.

Zacks *et al*.^27^ present a theoretical argument called Event Segmentation Theory (EST) which proposes that event perception involves segmenting narratives into boundaries based on prediction error. As comprehenders view visual sequences, they form predictions about what will happen next. When those predictions are disconfirmed (a prediction error), the viewer perceives this as a boundary in the narrative event. In a series of follow-up experiments, Zacks *et al*.^26^ presented participants with movies of everyday events. The movies were paused approximately once per minute and participants were asked to predict what would happen five seconds later in the film. They found that participants provided less accurate predictions, slower response times, and lower confidence in their predictions when the movies were stopped at an event boundary compared to in the middle of an event. In other words, it is harder to predict near-future events when one film scene has just ended and the other has not yet begun, compared to in the middle of an ongoing scene. Although this work clearly highlights the importance of prediction in visual event comprehension, none of these studies by Zacks and colleagues actually manipulate predictability by, for example, including predictable or unpredictable events or classifying stimuli based on cloze ratings.

In a series of experiments, Cohn & Paczynski^25^ explored the “Agent advantage” in visual narratives: the phenomenon that, when describing an event, people tend to mention the actor (or ‘Agent’) who has initiated the action before the undergoer (or ‘Patient’) who has received the action. In their Experiment 2, participants were presented with single comic panels depicting either an Agent or a Patient in a preparatory state and asked to describe “what happens next”? For instance, a panel of a character wearing a boxing glove with their fist pulled back might be expected to generate predictions that they were about to punch someone or something. They found that Agents in preparatory states generated more accurate predictions (i.e. participants’ predictions more often aligned with what was depicted in the original narrative sequence) than Patients in preparatory states, suggesting that these characters can facilitate predictions about upcoming actions. Although this work provides valuable insight into how comprehenders make predictions about upcoming events, like the work by Zacks and colleagues, it did not specifically manipulate predictability by comparing predictable and unpredictable events.

Predictability was specifically manipulated in an ERP study by Reid & Striano^23^, who presented participants with videos of an actress performing three different goal-directed actions: inserting a spoon into her mouth; putting on shoes; and answering a telephone. In addition to the predictable goal-directed actions, the video sequences also included “unpredictable” actions such as putting an empty spoon into the mouth, putting on only one shoe, or answering the phone by placing it on top of the head. The authors reported no significant differences between predictable and unpredictable actions for the shoe and telephone actions, but a larger N400 to unpredictable actions compared to predictable actions for the eating sequences. The authors attributed the lack of significant effects in two of the three conditions to factors such as spatial ERP smearing between participants, anticipatory processes due to the repeated presentations of the stimuli, or fundamental differences between putting on shoes/answering the phone compared to eating, with the latter having more concrete intentions. Nevertheless, all of the “unpredictable” actions in this study could be viewed as semantic anomalies (e.g. putting an empty spoon in the mouth) which were semantically implausible in addition to being unpredictable. This is an important distinction to make, since in the psycholinguistic literature it is clear that predictability (expected/unexpected) and semantic congruity (plausible/implausible) are separable effects: words can be unexpected but still semantically congruent^29^, and just the manipulation of expectedness (in the absence of semantic anomalies) is enough to elicit N400 differences^11,30^. We would therefore argue that because the Reid & Striano study included only semantically congruent and incongruent actions, they did not manipulate predictability (by, for example, including sequences that were unexpected but still plausible), and they included no quantifiable measurement of such “predictability.” Therefore, it remains unclear whether plausible events that are more or less predictable (e.g. high versus low cloze) would modulate the N400 for visual sequences in the same way as in language. Furthermore, Reid & Striano only included three different video sequences that were presented multiple times, meaning that an investigation of how predictability modulates N400 amplitude at the item level could not be performed. It thus remains unknown whether predictability would modulate the N400 for visual sequences in a continuous and graded way (i.e. would inversely correlate with N400 amplitude), as observed in language^31^.

The current study aims to quantify predictability using cloze ratings and systematically investigate how visual narrative sequences that are more or less predictable, yet still semantically plausible, modulate the N400 effect. Predictability in visual narratives can be tested using the same type of paradigm as in language studies, in which a target panel is more or less expected based on the prior narrative context. Here we examined the N400s to short visual narratives of comic strips, which, like sentences, unfold across a sequence with predictable or unpredictable outcomes. Notably, and in contrast to previous studies examining predictability in visual narrative sequences, we obtain quantifiable cloze ratings based on a previous norming study, as is the typical procedure in psycholinguistic studies of cloze. By quantifying and manipulating cloze in this way, we aim to demonstrate that predictability in visual narrative sequences modulates the N400 response in similar ways as in language, thereby providing further evidence that visual and linguistic narrative processing rely on similar cognitive mechanisms. We expect that low-cloze stimuli overall will show larger N400 amplitudes compared to high cloze stimuli. Furthermore, we expect this effect to be continuous: that is, cloze rating will be positively correlated with amplitude (i.e. as cloze rating goes up, or becomes more predictable, N400 amplitude will go up, or become less negative). As the N300 component has been more associated with object categorization or identification^14,15^, we do not expect this component to be modulated by cloze.

## Materials and Methods

### Participants

Participants were 22 typically developing adults ages 18–50 (*M* = 26, *SD* = 9.3; 17 female, 5 male) without any histories of neurological disorders or learning disabilities. The data from two additional participants were not usable and were not included in analyses. Using a repeated-measures ANOVA with 3 conditions, this sample size allows us to detect a medium-to-large effect size at a power of 0.80 and an alpha of 0.05 (see Supplementary Methods), similar to a previous ERP paradigm using visual narratives^24^. Participants were recruited from the University of Vermont and the broader Burlington, Vermont community through newspaper advertisements, fliers, and public announcements. All procedures were approved by the Institutional Review Board at the University of Vermont and were performed according to the guidelines and regulations of this committee. We obtained written informed consent from each participant before experimental testing. Participants were monetarily compensated for their time.

All participants self-reported normal or corrected-to-normal vision. Two participants were left-handed. (We also ran all statistical analyses with handedness as a covariate and the results were identical.) Participants completed the Visual Language Fluency Index (VLFI) which assesses expertise with visual narratives (*M* = 7, range 2–15). The total score on this questionnaire correlates with metrics of visual narrative comprehension^32,33^. A VLFI score of 12 is considered “average” comic reading expertise, with <8 being considered “low” and >22 considered “high” fluency^34^. On the VLFI participants also confirmed and rated their familiarity with *Peanuts* comics. All participants rated themselves as being familiar with the *Peanuts* comics. Although this familiarity could have influenced predictability mechanisms, as described in the next section, the strips used here were created by recombining panels of original *Peanuts* comics and, in some cases, unique panels were created to form fully novel strips. This renders it virtually impossible for participants to predict upcoming events in these sequences based on prior familiarity with these specific *Peanuts* comics, though common themes may have prevailed. We also note that “familiarity” may be vaguely interpreted, since the *Peanuts* franchise comprises different media (e.g., participants may know the characters or have seen the movies, but never read the original comic strips).

### Stimuli

**Figure 1:**
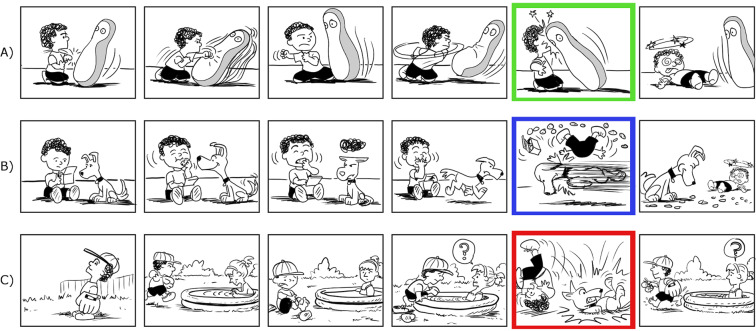
Examples of high-cloze (**A**) and low-cloze (**B**) visual narrative stimuli. Bolded boxes indicate the critical panel in each sequence. In high-cloze stimuli, the critical panel is highly predictable given the prior context; in low-cloze stimuli, the critical panel is less predictable. Panel (**C**) shows an example of an anomalous sequence, in which the critical panel is completely semantically incongruent with the preceding context.

We selected stimuli from a corpus of over 500 visual narrative sequences recombined from panels of Charles Schulz’s *The Complete Peanuts* (Fantagraphic Books, 2004–2006) to create novel experimental sequences as used in several prior studies^32,34^. We obtained cloze ratings for 265 sequences during a pre-test with 200 healthy adults who were not included in the main experiment. Stimuli were presented via Survey Monkey. Each 6-panel sequence was cropped just before the climactic “Peak” panel^35^, which occurred between positions 3 and 6, depending on the sequence. These cropped sequence stems, which varied from 2 to 5 panels long, were presented to participants who were asked “what comes next?” They typed descriptions of their predicted events at the next panel into a text box. Answers were coded based on their reference to topical statements capturing the main idea of what would happen next, regardless of the specific linguistic utterances. See Supplementary Table S1 for example responses. For example, in statements about the outcome of the sequence in Fig. 1A, responses of “The bopper hits the kid”, “The punching bag knocks him over,” and “Bop-bag comes back up and hits him” were all considered as matches for their shared gist of “being hit by the toy”, despite using different phrasing. Some examples had multiple agreed-upon outcomes, or variations on the main idea that were associated but not an exact match. For the purposes of calculating cloze ratings for the current study, we used only the answers that were categorized as exact matches, making our agreement criteria fairly restrictive.

The predicted responses for each sequence across all participants were then collated to determine cloze ratings. Cloze ratings for each sequence corresponded to the proportion of participants who agreed on the ending (ranging from 0–1). Thus, if 100 of the 200 respondents (50%) had agreed upon the outcome depicted in the subsequent Peak panel, that sequence would have a cloze rating of 0.5. Using these cloze ratings, we selected 60 sequences with high cloze Peak (critical) panels (>0.4, *M* = 0.6; see Fig. 1A for an example) and 60 with low cloze Peak panels (<0.25, *M* = 0.2; Fig. 1B). In some cases, novel panels were created to fit the “best completion” ending described by respondents for high-cloze strips. An additional 60 sequences served as anomalous stimuli (Fig. 1C) by replacing Peak panels (which had original cloze ratings ranging from 0 to 0.83, *M* = 0.4) with semantically incongruous panels from different sequences. The constraint ratings for anomalous strips (i.e. the degree to which the preceding narrative context biases the prediction of a certain event^7^) also ranged from 0 to 0.83 (*M* = 0.40).

### Procedure

Participants attended one testing session (approximately two hours) which comprised of consenting and paperwork, behavioral tests, application of the EEG net, and experimental testing. Stimuli were presented using E-Prime version 2.0.10.356. The 180 total stimuli were presented as 6 blocks of 30 stimuli. Each block contained an equal number of sequences from each condition, presented in pseudorandom order. Before each trial a “Ready?” screen was presented until participants pressed a button, followed by a fixation cross for 500 ms. Panels were presented one at a time in the center of the screen for 1350 ms each with an ISI of 350 ms^33^.

A subset of trials (20%) included a comprehension question. After each sequence, an additional panel appeared in the center of the screen, marked as a comprehension question by a red question mark above the panel and a red border around the slide. Participants indicated whether the panel was related to the sequence they had just seen. Comprehension questions were evenly distributed across conditions and blocks, and contained an equal number of ‘related’ and ‘unrelated’ correct answers. The comprehension questions emphasized accuracy rather than speed.

Stimuli were presented on a Dell 21.5” LCD monitor with 1920 × 1080 resolution. Participants sat in a comfortable chair in a quiet room separate from the experimenter and computers. Participants sat approximately 24” away from the screen and each comic panel was 5.25” wide and 4.125” high, yielding a visual angle of 12.56° horizontally and 9.8° vertically.

### EEG data acquisition and analysis

EEG data were recorded continuously at 500 Hz using EGI’s Geodesic EEG System (GES) 400, a 128-channel EGI Geodesics Sensor net, and NetStation version 5.4. A 4 kHz antialiasing lowpass filter was applied during acquisition and data were referenced online to the Cz electrode. Impedances were kept under 50 kΩ wherever possible. EEG data preprocessing was performed with EEGlab version 14.1.1b^36^ and Matlab 2017a. The data were filtered using a 0.1–50 Hz bandpass filter. Epochs were time-locked to the onset of the critical (Peak) panel in each sequence and extended from 100 ms before to 1500 ms after the critical panel. Independent component analysis (ICA)^37,38^ was used for artifact correction. Before ICA decomposition the mean of each trial was removed^39^ and data were reduced to 32 dimensions. After ICA decomposition the ERP waveforms and topographic plots of each component were reviewed and a trained examiner marked any components contributing to movement, eye blinks or saccades, or other sources of noise for removal. Segments were then baseline corrected using the first 100 ms of the segment and re-referenced to the average of the left and right mastoids. Additional bad trials were identified and rejected using a joint probability computation^38^ with a threshold of 3 standard deviations. Finally, each trial was visually reviewed and any further bad trials not caught by the joint probability algorithm were marked for removal by a trained experimenter. (See Supplementary Figure S1.) On average, 74% of trials (44 per trial type) were included in the statistical analyses (anomalous = average 45/60 trials retained, low cloze = 44/60 trials; high cloze = 43/60 trials). See Supplementary Table S2 for details on the number of trials included for each participant.

### Statistical analysis

**Figure 2:**
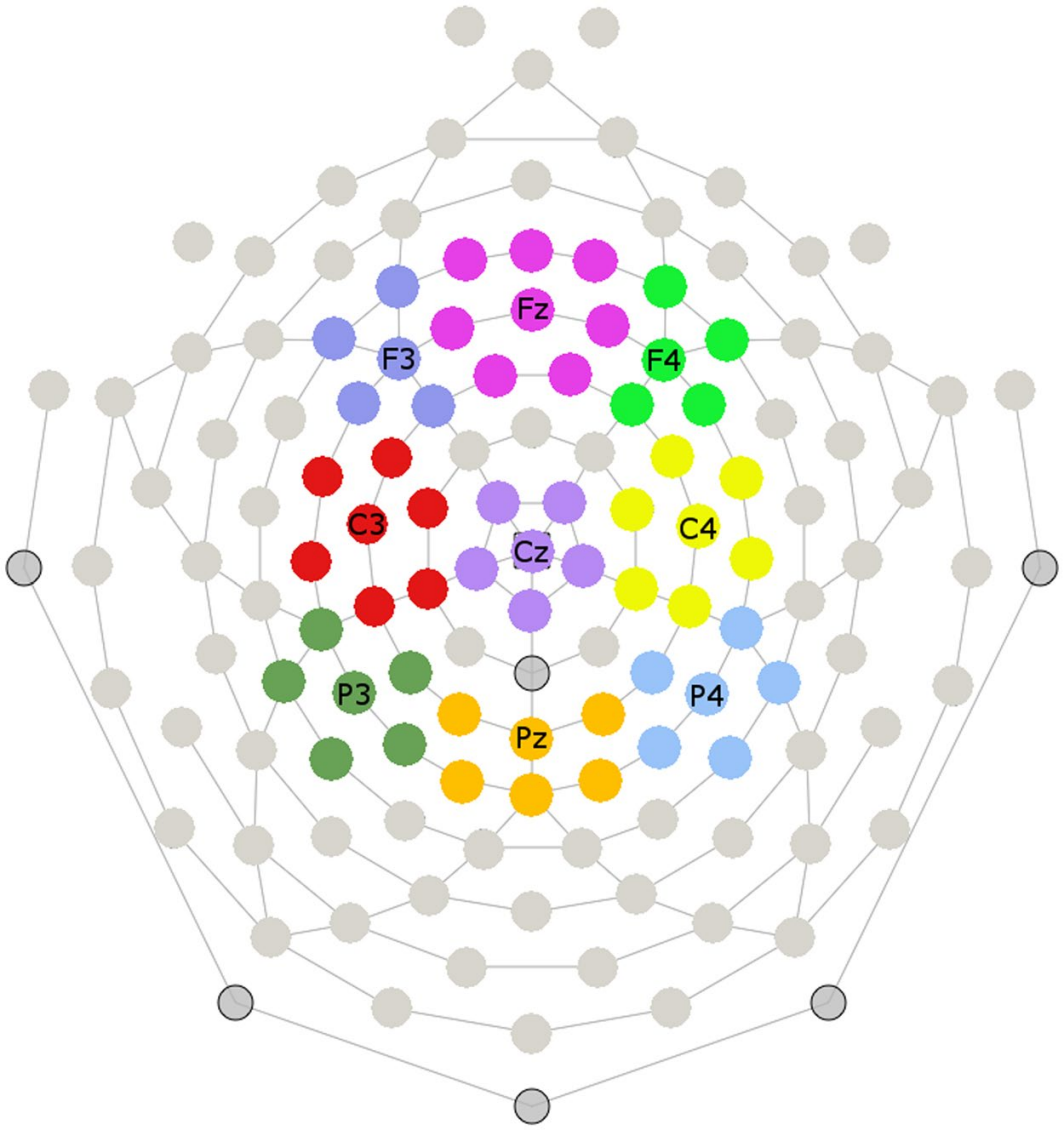
Illustration of the nine electrode clusters used for EEG analysis, with centers of each cluster labeled.

Statistical analyses were performed in R version 3.4.1 (R Core Team, 2015). ERP amplitude was evaluated at nine electrode clusters centered based on the 10–20 distribution (F3, Fz, F4, C3, Cz, C4, P3, Pz, and P4^24,40^; Fig. 2). Each cluster contained between 5 and 7 electrodes. We have used this clustered electrode distribution in previous studies^24,40^ to provide a broad scalp representation and ensure we did not miss potential effects outside of a predefined analysis region, since visual narratives are known to elicit N400 effects at slightly different scalp topographies than linguistic stimuli^22,24,32^. Time windows for analyses were based on visual inspection and included windows from 200–300, 400–500, 500–600, 600–800, and 800–1000 ms (out of the full epoch of 0–1500 ms with a 100 ms prestimulus baseline period). To evaluate effects of condition on ERP amplitude, repeated-measure ANOVAs were run with within-subject factors of *condition* (anomalous/low cloze/high cloze), *site* (frontal/central/parietal), and *laterality* (left/midline/right). Full results are shown in Table 1. Only significant effects of, or interactions with, condition are reported in the text. Post hoc paired-sample *t*-tests were used to examine significant effects of condition. Generalized eta-squared (*η*²_G_) and Cohen’s *d* are reported as measures of effect sizes for ANOVAs and *t*-tests, respectively. Finally, to examine whether the effects of predictability elicited continuous modulations of ERP amplitude, cloze ratings for each visual narrative sequence in the high and low cloze conditions were correlated with amplitude at every timepoint (downsampled to 250 Hz) from 200–1000 ms at each cluster and in the time window showing significant differences in the overall ANOVA. Cluster mass permutation tests were used to account for multiple comparisons in the full timecourse analysis.

**Table 1:** *F*-values for the repeated-measures ANOVAs in each analysis window. Within-subjects factors are *condition* (high cloze, low cloze, anomalous), *site* (frontal, central, parietal), and *laterality* (left, midline, right). Effect size (*η*²_G_) is indicated in parentheses. Asterisks indicate statistically significant results (*p < 0.05; **p < 0.01; ***p < 0.001). Main effects of condition and interactions with condition are highlighted in bold.

Main effect or interaction	Time windows				
	200–300 ms	400–500 ms	500–600 ms	600–800 ms	800–1000 ms
**Condition**	1.29 (<0.01)	1.42 (<0.01)	2.88 (0.02)*	2.61 (0.02)	1.28 (0.01)
Site	34.67 (0.32)***	14.48 (0.15)***	12.39 (0.12)***	8.47 (0.08)***	7.51 (0.05)***
Laterality	5.40 (0.02)**	3.43 (<0.01)*	3.17 (<0.01)	1.38 (<0.01)	0.54 (<0.01)
Condition*site	0.57 (<0.01)	8.73 (<0.01)***	6.78 (<0.01)**	2.97 (<0.01)*	1.63 (<0.01)
Condition*laterality	2.91 (<0.01)*	0.46 (<0.01)	0.84 (<0.01)	1.27 (<0.01)	3.02 (<0.01)*
Site*laterality	9.99 (0.01)***	1.66 (<0.01)	0.96 (<0.01)	0.58 (<0.01)	0.42 (<0.01)
Condition*site*laterality	4.71 (<0.01)***	1.65 (<0.01)	1.00 (<0.01)	0.81 (<0.01)	0.60 (<0.01)

## Results

### Behavioral results

We first confirmed that participants were all attending to and understanding the narratives by evaluating behavioral performance on the comprehension questions. A 3-way ANOVA showed a main effect of condition (*F*(2,42) = 8.37, *p* < 0.001) such that accuracy was significantly lower for anomalous conditions (*M* = 81%) compared to the other two conditions (low cloze *M* = 89%; high cloze *M* = 87%; all *p*’s < 0.05), whereas the high and low cloze conditions did not differ (*p* = 0.28). All three conditions had significantly higher accuracy rates compared to chance (50%), as determined by one-sample *t*-tests (all *p*’s < 0.0001). This suggests that participants were able to successfully understand the narratives.

### ERP results

**Figure 3:**
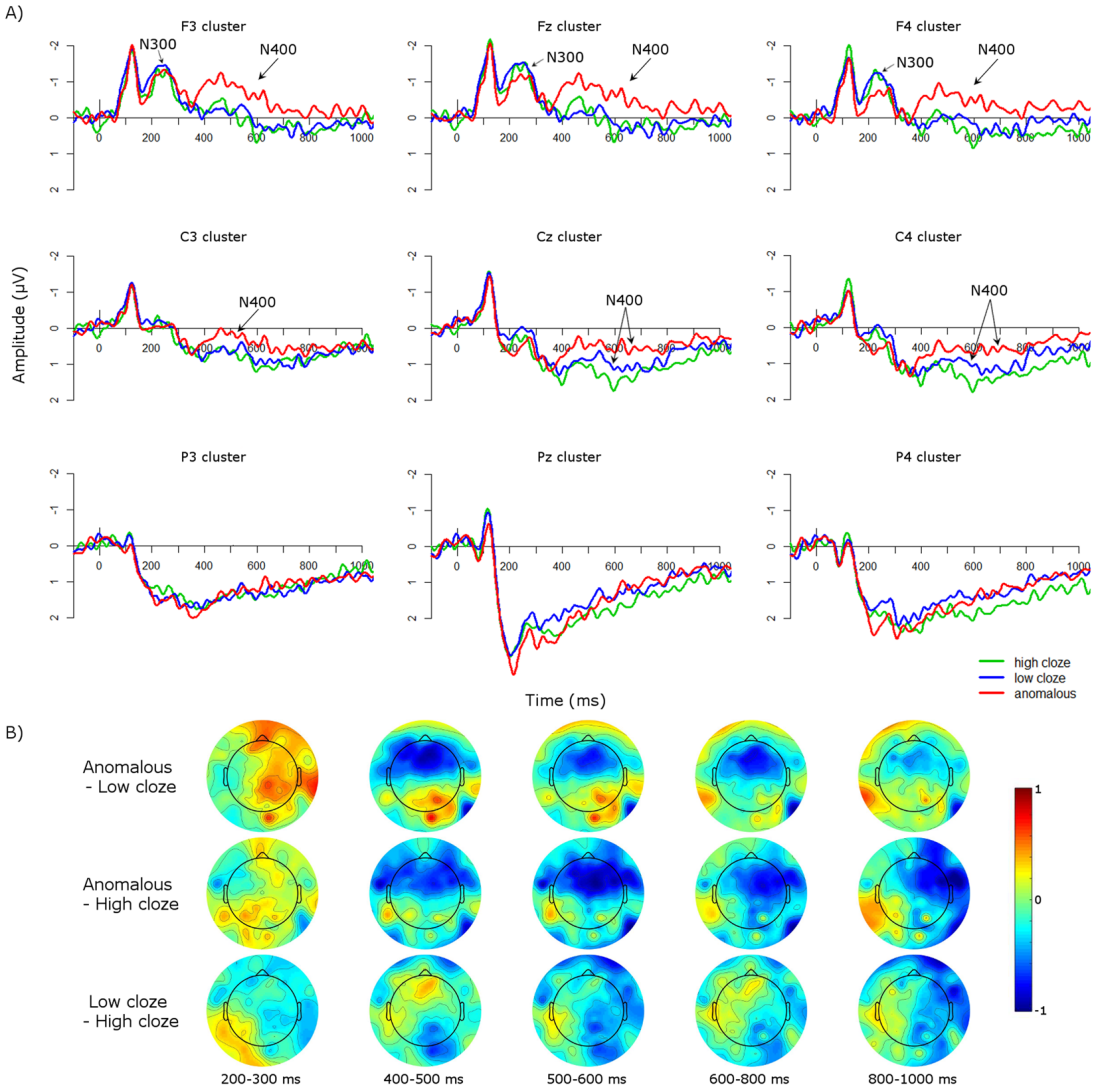
(**A**) ERPs at the critical panel for each condition at nine clusters across the scalp. Negativity is plotted upwards. Data is filtered using a 30 Hz lowpass filter for presentation purposes only. N300 and N400 effects are labeled. (**B**) Topographic plots of the three comparisons of interest across the five time windows of interest.

Visual inspection of the ERP waveforms, depicted in Fig. 3, suggested an early negativity, particularly over frontal scalp, from approximately 200–300 ms, identified as an N300 component. Following the N300 component there was an enhanced negative amplitude for the anomalous condition compared to high and low cloze conditions from approximately 400–800 ms, particularly over frontal and central scalp. Finally, the high cloze and low cloze conditions appeared to diverge between 500 and 600 ms and between 800–1000 ms. To evaluate these differences statistically, we ran repeated-measures ANOVAs from 200–300, 400–500, 500–600, 600–800, and 800–1000 ms.

#### 200–300 ms

A significant interaction occurred between condition, site, and laterality (*F*(8,168) = 4.71, *p* < 0.001, *η*²_G_ = 0.0006; Table 1). To explore this interaction, we ran ANOVAs at each laterality with factors of condition and site. There was a significant condition by site interaction in the left hemisphere only (*F*(4,84) = 2.76, *p* < 0.05, *η*²_G_ = 0.003). However, follow-up testing with one-way ANOVAs at each site with condition as a factor revealed no main effects of condition at any site, suggesting no effects of condition on the N300 component.

#### 400–500 ms

A significant interaction occurred between condition and site (*F*(4,84) = 8.73, *p* < 0.001, *η*²_G_ = 0.007; Table 1). To explore this interaction, we ran one-way ANOVAs at each site with condition as a factor. There was a main effect of condition at frontal sites (*F*(2,42) = 3.96, *p* < 0.05, *η*²_G_ = 0.02) only. Follow-up paired-samples *t*-tests comparing conditions at frontal sites indicated that anomalous conditions were significantly more negative than high cloze (*t*(65) = 3.39, *p* < 0.01, *d* = 0.42) and low cloze (*t*(65) = 3.67, *p* < 0.001, *d* = 0.45) conditions, but high and low cloze did not differ (*p* = 0.19).

#### 500–600 ms

A significant interaction occurred between condition and site (*F*(4,84) = 6.78, *p* < 0.01, *η*²_G_ = 0.006; Table 1). To explore this interaction, we ran one-way ANOVAs at each site with condition as a factor. There was a main effect of condition at frontal (*F*(2,42) = 5.56, *p* < 0.01, *η*²_G_ = 0.03) and central sites (*F*(2,42) = 3.39, *p* < 0.05, *η*²_G_ = 0.03). At frontal sites, paired-sample *t*-tests indicated that the anomalous condition was significantly more negative than high cloze (*t*(65) = 4.51, *p* < 0.0001, *d* = 0.56) and low cloze (*t*(65) = 3.63, *p* < 0.001, *d* = 0.45) conditions, but high and low cloze did not differ (*p* = 0.44). At central sites, the anomalous condition was significantly more negative than high cloze (*t*(65) = 3.50, *p* < 0.001, *d* = 0.43) and low cloze (*t*(65) = 2.10, *p* < 0.05, *d* = 0.26), and while low cloze was more negative than high cloze (*t*(65) = 3.03, *p* < 0.01, *d* = 0.37).

#### 600–800 ms

A significant interaction occurred between condition and site (*F*(4,84) = 2.97, *p* < 0.05, *η*²_G_ = 0.002; Table 1). To explore this interaction, we ran one-way ANOVAs at each site with condition as a factor. There was a main effect of condition at frontal sites (*F*(2,42) = 3.75, *p* < 0.05, *η*²_G_ = 0.03) only. Paired-sample *t*-tests between conditions at frontal sites indicated that the anomalous condition was significantly more negative than high cloze (*t*(65) = 3.61, *p* < 0.001, *d* = 0.44) and low cloze (*t*(65) = 3.43, *p* < 0.01, *d* = 0.42), but high and low cloze did not differ (*p* = 0.98).

#### 800–1000 ms

A significant interaction occurred between condition and laterality (*F*(4,84) = 3.02, *p* < 0.05, *η*²_G_ = 0.003; Table 1). To explore this interaction, we ran one-way ANOVAs at each laterality with condition as a factor. There was a trend of a main effect of condition at right-hemisphere sites (*F*(2,42) = 3.00, *p* = 0.06, *η*²_G_ = 0.03). Paired-sample *t*-tests between conditions at right-hemisphere sites indicated that the high cloze condition was significantly more positive than the anomalous (*t*(65) = 4.51, *p* < 0.001, *d* = 0.55) and low cloze conditions (*t*(65) = 2.59, *p* < 0.05, *d* = 0.31), but there were no differences between anomalous and low cloze conditions (*p* = 0.26).

To summarize, the anomalous condition showed a significant N400 effect, with enhanced negative amplitudes compared to both low and high cloze conditions from 400–800 ms over fronto-central electrodes. The high cloze and low cloze conditions also diverged between 500 and 600 ms, such that low cloze sequences were more negative than high cloze sequences over central scalp.

### Correlations with cloze

**Figure 4:**
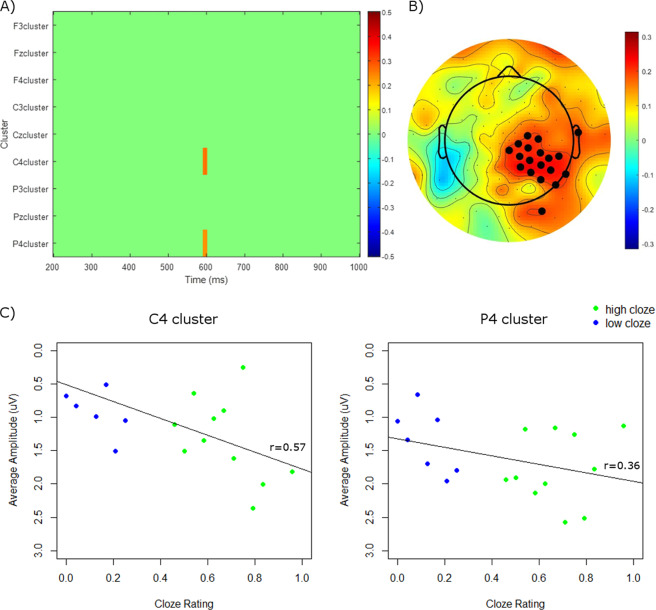
Correlations of cloze rating with amplitude. (**A**) Correlations of cloze rating with amplitude at each timepoint and electrode cluster. Correlation coefficients are plotted as a heat map with positive correlations in red. Non-significant correlation coefficients (as determined by cluster mass permutation tests to account for multiple comparisons) are masked in green. (**B**) Topographic plot of *r* correlation coefficients between cloze rating and amplitude from 500–600 ms at every electrode over the scalp. Black dots indicate electrodes where *p* < 0.05. (**C**) Scatterplot of cloze rating with amplitude at the C4 (left) and P4 (right) clusters from 500–600 ms. Negative is plotted upwards.

To further examine the relationship between cloze and N400 amplitude, we ran three different sets of correlations. First, we correlated cloze ratings for each visual narrative sequence in the high cloze or low cloze conditions with amplitude at every timepoint from 200–1000 ms (downsampled to 250 Hz, i.e. every 4 ms) at each cluster. Corrections for multiple comparisons were performed using cluster mass permutation tests^41,42^. The results are plotted as a spectral plot in Fig. 4A. As can be seen, cloze rating correlates positively with amplitude over midline and right centro-parietal scalp.

Second, we correlated cloze with amplitude from 500–600 ms (the window in which the N400 ERP amplitude showed differences between high and low cloze conditions) at every electrode across the scalp. As can be seen in Fig. 4B, which shows a topographic plot of the *r* correlation coefficients across the scalp, there was a cluster of significant (*p* < 0.05) correlations over right centro-parietal scalp.

Third, we correlated cloze with amplitude over the 500–600 ms window at two selected electrodes, C4 and P4 (based on findings from our ANOVAs and the previous two correlation methods suggesting that the correlations with cloze were strongest at right-lateralized central and parietal scalp). Items were binned over cloze ratings, as has been done in previous studies^7,43^. This relationship is depicted in Fig. 4C, which show scatterplots of cloze rating versus average amplitude from 500–600 ms at the C4 and P4 clusters. Although a significant correlation was observed at the C4 cluster (*r* = 0.57, *p* < 0.05), the correlation at the P4 cluster did not reach significance (*r* = 0.36, *p* = 0.14). (When running the correlations with every item included, the opposite pattern occurred, with a significant correlation at the P4 cluster (*r* = 0.20, *p* < 0.05), but not at the C4 cluster (*r* = 0.15, *p* = 0.12); see Supplementary Material S2. This switch in effects at different electrodes may be a result of individual variability in the amplitudes at central and parietal clusters. As can be seen in the scatterplot of the full item analysis, presented in Supplementary Material S2, even among items with the same cloze rating there is a fair amount of spread in the magnitude of N400 amplitudes. In the binned analysis, presented in Fig. 4C, items with the same cloze rating are averaged together, which collapses this spread into a single point per bin.)

Across all three of these analyses the correlations were positive, indicating that as cloze rating decreases (i.e. as sequences become less predictable), amplitude decreases (i.e. becomes more negative).

## Discussion

This study tested the hypothesis that understanding narrative sequences of images, like understanding sequences of words in sentences, is modulated by the predictability of upcoming information. To do so we employed a cloze probability ERP paradigm in which a critical panel in a visual narrative sequence was either more or less predictable given the preceding context of the story. We examined N400 ERP responses to three types of sequences: high cloze, low cloze, and anomalous. If visual narrative processing relies on similar cognitive mechanisms as language^31^, we would expect visual narratives to elicit larger N400 amplitudes for low cloze stimuli compared to high cloze stimuli, as is found in the language literature, with N400 amplitudes for anomalous stimuli being the largest of the three conditions.

We did indeed observe this expected pattern. The anomalous images evoked more negative amplitudes than the high and low cloze conditions from 400–800 ms over fronto-central scalp. This replicates previous findings that semantic violations in narrative comprehension elicit large N400 responses as a result of trying to process unpredictable semantic information^22,24,32^. However, our main finding of this study is that the low cloze conditions were also more negative than the high cloze conditions from 500–600 ms over central scalp. These results demonstrate that N400 amplitude is sensitive to cloze probability in visual narrative sequences, just as in linguistic stimuli. We acknowledge that this effect was small, being observed in a relatively short time window (500–600 ms) and with a small-to-moderate effect size (Cohen’s *d* = 0.37). Nevertheless, this finding offers support for the common sensitivity to predictability underlying comprehension across domains.

Our second main finding of this study is that we observed small but significant correlations of cloze rating with amplitude across high and low cloze items. More specifically, ERP amplitude was positively correlated with cloze ratings in a graded, non-discrete way, such that larger N400 amplitudes (i.e. increasing negativities, decreasing amplitudes) were associated with less predictable stimuli (i.e. lower cloze ratings). This association indicates that for less predictable sequences, comprehenders must work harder to integrate incoming information with the unfolding narrative representation, resulting in larger negativities. These correlations were modest (*r*-value of 0.57 at the C4 cluster, corresponding to a small correlation^44^), and in the full timecourse analysis appeared in a narrow time window (approximately 590–600 ms), likely as a result of the stricter correction for multiple comparisons. Nevertheless, taken together, these results do suggest that cloze modulates ERP amplitude in a continuous way, as in language^7^. Interestingly, the correlations of amplitude with cloze ratings showed slightly different scalp distributions than effects observed in the overall ANOVA. Over all methods of correlational analyses, correlations were strongest over the right hemisphere at central and parietal sites (C4 and P4 clusters), whereas significant modulations of N400 amplitude by condition occurred only at central sites and were not lateralized to one hemisphere. These correlations may be picking up more subtle modulations of amplitude by cloze rating which are not captured when items are grouped together into high or low cloze categories or over larger analysis windows. In any case, the small but significant associations of cloze ratings and negative amplitudes replicate previous findings in the linguistic domain^12^.

As we have emphasized above, the differences between high and low cloze in the ANOVA, and the correlations between cloze and N400 amplitudes, were small. This is in contrast to the large and robust effects of cloze that are often observed in linguistic studies^7^ and points to the need for further confirmatory analyses to replicate this finding. One possibility is that our assessment of cloze probability was fairly restrictive, in that our agreement scores looked for “exact matches” of the gist of upcoming information (see examples in Supplementary Table S1). This meant that statements were deemed as “not agreeing” if they over-specified or elaborated on a similar idea of what happened next, resulting in a fairly restrictive assessment of predictability, resulting sometimes in lower cloze probability scores.

In turn, our high cloze condition included items with cloze ratings of <0.4, with a mean of 0.6. This is a lower cutoff compared to many linguistic studies (e.g. the high-cloze stimuli used in DeLong *et al*.^29^ had a mean cloze of 0.88). This could have introduced variability in the N400 response that mitigated the overall difference between high and low cloze. However, over all high and low cloze items, our cloze ratings ranged from 0 to 0.96, meaning that our correlational analyses (which considered all items rather than binning them into high/low cloze) should have been able to identify the full range of cloze effects. We did indeed observe significant correlations of cloze with amplitude, although again these effects were small and require further replication. Despite the small effect sizes in the current study, this work is notable given the limited literature systematically manipulating predictability in visual narratives. As discussed earlier, although predictability has been discussed in terms of how comprehenders segment narratives into event boundaries^25–27^, there has been only one study that directly compared predictable and unpredictable events within a visual event sequence^23^. However, as we argue above, this prior study actually compared anomalous events to predictable events, which in essence only replicates the known anomaly effect on the N400^7^. Here we manipulate predictability using quantifiable metrics of expectedness based on cloze ratings, thereby including graded variations in predictability. By modeling our cloze probability paradigm for visual narratives on the well-established paradigm in linguistic research, we demonstrate that predictability in visual narratives modulates the N400 effect in a graded, continuous way. The similarities of predictability effects between our results and those documented in the linguistics literature^7,29^ serve to further demonstrate the similar cognitive mechanisms underlying narrative comprehension processes across domains.

Of note, the participants tested here were not recruited on the basis of comic-reading fluency and, indeed, showed low-to-average levels of comic-reading expertise (VLFI scores ranging from 2–15, average of 7). In a post-hoc analysis we also correlated VLFI scores with the N400 effect between high cloze and low cloze conditions from 500–600 ms, but no significant correlations emerged (all *p*’s < 0.22). This suggests that modulation of the N400 effect by predictability does not depend on participants’ experience with comic reading, consistent with prior observations that comics reading expertise does not modulate the N400, despite modulation of other ERP components^45^.

It is also worth noting that the timing of N400 effects observed here is slightly later than typical N400 effects documented in the language literature. N400 effects for high or low cloze words in sentences generally elicit N400 effects from approximately 200–500 ms^3,29^. Here, we observed larger N400 amplitudes for anomalous conditions compared to low and high cloze from 400–800 ms, and between low and high cloze from 500–600 ms. These later time windows may be reflective of the more complex visual information inherent in these stimuli. The timing of these effects are, however, consistent with previous studies using visual sequences which have shown slightly later and more sustained negativities resulting from semantic processing and integration^22,24,32,46^. In a previous study of predictable and unpredictable visual action sequences, Reid & Striano^23^ also found modulation of the N400 effect in a slightly later window from 500–700 ms. The fronto-central distribution of the N400 effects observed here is also consistent with prior studies using visual narratives, which have shown more anterior negativities, in contrast to the more parietal N400 distributions for linguistic stimuli^7^. Therefore, although we observed slight differences in scalp distribution and timing of the predictability effect on ERP amplitude, the underlying cognitive mechanism of how predictability influences narrative comprehension appears to be similar between visual and linguistic narratives.

In sum, these results demonstrate that the N400 ERP component is sensitive to cloze probability in visual narratives, just as in linguistic narratives. These data offer further evidence that sequential images are processed using similar cognitive and neural mechanisms as language^31^, and provide further support for the domain generality of semantic processing in the brain.

## Supplementary information

Supplementary information.

## References

[1] Federmeier KD (2007). Thinking ahead: The role and roots of prediction in language comprehension. Psychophysiology.

[2] Venker CE, Edwards J, Saffran JR, Ellis Weismer S (2019). Thinking Ahead: Incremental Language Processing is Associated with Receptive Language Abilities in Preschoolers with Autism Spectrum Disorder. J. Autism Dev. Disord..

[3] Ito A, Martin AE, Nieuwland MS (2017). How robust are prediction effects in language comprehension? Failure to replicate article-elicited N400 effects. Lang. Cogn. Neurosci..

[4] Rabovsky M, McRae K (2014). Simulating the N400 ERP component as semantic network error: Insights from a feature-based connectionist attractor model of word meaning. Cognition.

[5] Kuperberg GR (2016). Separate streams or probabilistic inference? What the N400 can tell us about the comprehension of events. Lang. Cogn. Neurosci..

[6] Martin CD (2013). The impact of early bilingualism on controlling a language learned late: An ERP study. Front. Psychol..

[7] Kutas M, Federmeier KD (2011). Thirty years and counting: Finding meaning in the N400 component of the event-related brain potential (ERP). Annu. Rev. Psychol..

[8] Kutas M, Hillyard S (1980). Reading Senseless Sentences: Brain Potentials Reflect Semantic Incongruity. Science (80-.)..

[9] Brouwer H, Fitz H, Hoeks J (2012). Getting real about Semantic Illusions: Rethinking the functional role of the P600 in language comprehension. Brain Res..

[10] Lau EF, Phillips C, Poeppel D (2008). A cortical network for semantics: (de)constructing the N400. Nat. Rev. Neurosci..

[11] Kutas M, Hillyard S (1984). Brain potentials during reading reflect word expectancy and semantic association. Nature.

[12] Van Petten C, Luka BJ (2012). Prediction during language comprehension: Benefits, costs, and ERP components. Int. J. Psychophysiol..

[13] Ganis G, Kutas M, Sereno MI (1996). The search for ‘common sense’: an electrophysiological study of the comprehension of words and pictures in reading. J. Cogn. Neurosci..

[14] Hamm JP, Johnson BW, Kirk IJ (2002). Comparison of the N300 and N400 ERPs to picture stimuli in congruent and incongruent contexts. Clin. Neurophysiol..

[15] McPherson WB, Holcomb PJ (1999). An electrophysiological investigation of semantic priming with pictures of real objects. Psychophysiology.

[16] Sitnikova T, Holcomb PJ, Kiyonaga K, Kuperberg GR (2008). Two Neurocognitive Mechanisms of Semantic Integration during the Comprehension of Visual Real-world Events. 2Journal Cogn. Neurosci..

[17] Võ MLH, Wolfe JM (2013). Differential Electrophysiological Signatures of Semantic and Syntactic Scene Processing. Psychol. Sci..

[18] Mudrik L, Lamy D, Deouell LY (2010). ERP evidence for context congruity effects during simultaneous object-scene processing. Neuropsychologia.

[19] Coco MI, Araujo S, Petersson KM (2017). Disentangling stimulus plausibility and contextual congruency: Electro-physiological evidence for differential cognitive dynamics. Neuropsychologia.

[20] Ganis G, Kutas M (2003). An electrophysiological study of scene effects on object identification. Cogn. Brain Res..

[21] Draschkow D, Heikel E, Võ MLH, Fiebach CJ, Sassenhagen J (2018). No evidence from MVPA for different processes underlying the N300 and N400 incongruity effects in object-scene processing. Neuropsychologia.

[22] West WC, Holcomb PJ (2002). Event-related potentials during discourse-level semantic integration of complex pictures. Cogn. Brain Res..

[23] Reid VM, Striano T (2008). N400 involvement in the processing of action sequences. Neurosci. Lett..

[24] Coderre EL (2018). Visual and linguistic narrative comprehension in autism spectrum disorders: Neural evidence for modality-independent impairments. Brain Lang..

[25] Cohn N, Paczynski M (2013). Prediction, events, and the advantage of Agents: The processing of semantic roles in visual narrative. Cogn. Psychol..

[26] Zacks JM, Kurby CA, Eisenberg ML, Haroutunian N (2011). Prediction error associated with the perceptual segmentation of naturalistic events. J. Cogn. Neurosci..

[27] Zacks JM, Speer NK, Swallow KM, Braver TS, Reynolds JR (2007). Event Perception: A Mind/Brain Perspective Jeffrey. Psychol. Bull..

[28] Magliano JP, Dijkstra K, Zwaan R (1996). Generating Predictive Inferences While Viewing a Movie. 2Discourse Process..

[29] DeLong KA, Quante L, Kutas M (2014). Predictability, plausibility, and two late ERP positivities during written sentence comprehension. Neuropsychologia.

[30] Brothers T, Swaab TY, Traxler MJ (2017). Goals and strategies influence lexical prediction during sentence comprehension. J. Mem. Lang..

[31] Cohn, N. Your Brain on Comics: A Cognitive Model of Visual Narrative Comprehension. *Top. Cogn. Sci*. 1–35 (2019). 10.1111/tops.12421 PMC9328425 30963724

[32] Cohn N, Paczynski M, Jackendoff R, Holcomb PJ, Kuperberg GR (2012). (Pea)nuts and bolts of visual narrative: structure and meaning in sequential image comprehension. Cogn. Psychol..

[33] Cohn N, Maher S (2015). The notion of the motion: The neurocognition of motion lines in visual narratives. Brain Res..

[34] Cohn N, Bender P (2017). Drawing the line between constituent structure and coherence relations in visual narratives. J. Exp. Psychol. Learn. Mem. Cogn..

[35] Cohn, N. The visual language of comics: Introduction to the structure and cognition of sequential images. (Bloomsbury, 2013).

[36] Delorme A, Makeig S (2004). EEGLAB: an open source toolbox for analysis of single-trial EEG dynamics including independent component analysis. J. Neurosci. Methods.

[37] Jung TP (2000). Removing electroencephalographic artifacts by blind source separation. Psychophysiology.

[38] Delorme A, Sejnowski TJ, Makeig S (2007). Enhanced detection of artifacts in EEG data using higher-order statistics and independent component analysis. Neuroimage.

[39] Groppe DM, Makeig S, Kutas M (2009). Identifying reliable independent components via split-half comparisons. Neuroimage.

[40] Coderre EL, Chernenok M, Gordon B, Ledoux K (2017). Linguistic and Non-Linguistic Semantic Processing in Individuals with Autism Spectrum Disorders: An ERP Study. J. Autism Dev. Disord..

[41] Maris E, Oostenveld R (2007). Nonparametric statistical testing of EEG- and MEG-data. J. Neurosci. Methods.

[42] Groppe DM, Urbach TP, Kutas M (2011). Mass univariate analysis of event-related brain potentials/fields I: A critical tutorial review. Psychophysiology.

[43] DeLong KA, Urbach TP, Kutas M (2005). Probabilistic word pre-activation during language comprehension inferred from electrical brain activity. Nat. Neurosci..

[44] Taylor R (1990). Interpretation of the Correlation Coefficient: A Basic Review. J. Diagnostic Med. Sonogr..

[45] Cohn, N. Visual narrative comprehension: Universal or not? *Psychon. Bull. Rev*. **27**, 266–285 (2020). 10.3758/s13423-019-01670-1 PMC7093370 31820277

[46] Sitnikova T, Kuperberg G, Holcomb PJ (2003). Semantic integration in videos of real-world events: An electrophysiological investigation. Psychophysiology.

