# Supplementary material for: Predictability modulates neurocognitive semantic processing of non-verbal narratives
Source: Sci Rep. 2020 Jun 25;10:10326. doi: 10.1038/s41598-020-66814-z (PMC7316725; doi:10.1038/s41598-020-66814-z)
Supplement: Supplementary file 1 — Supplementary information. [file 41598_2020_66814_MOESM1_ESM.docx]

Supplementary Information for

PREDICTABILITY MODULATES NEUROCOGNITIVE SEMANTIC PROCESSING OF NON-VERBAL NARRATIVES

Emily L. Coderre, Elizabeth O’Donnell, Emme O’Rourke, and Neil Cohn

Supplementary Methods:

Additional information on power analyses:

We conducted our power analysis using G*Power. For a repeated-measures ANOVA, within factors with 3 repetitions (corresponding to the three conditions) and a correlation of 0.6 among the measures (which is a slight under-approximation of the correlations between the different conditions), a sample size of 23 would allow us to detect a medium effect size (Cohen’s f = 0.25) with 80% power and an alpha level of 0.05. Using the same parameters, a sample size of 10 would be required to detect a large effect size. Therefore we feel confident that our sample of 22 is adequate for detecting a medium-to-large effect size.

Supplementary Figure S1: Comparison of the three contrasts of interest a) before data was cleaned and b) after data was cleaned, in each time window used in analyses.


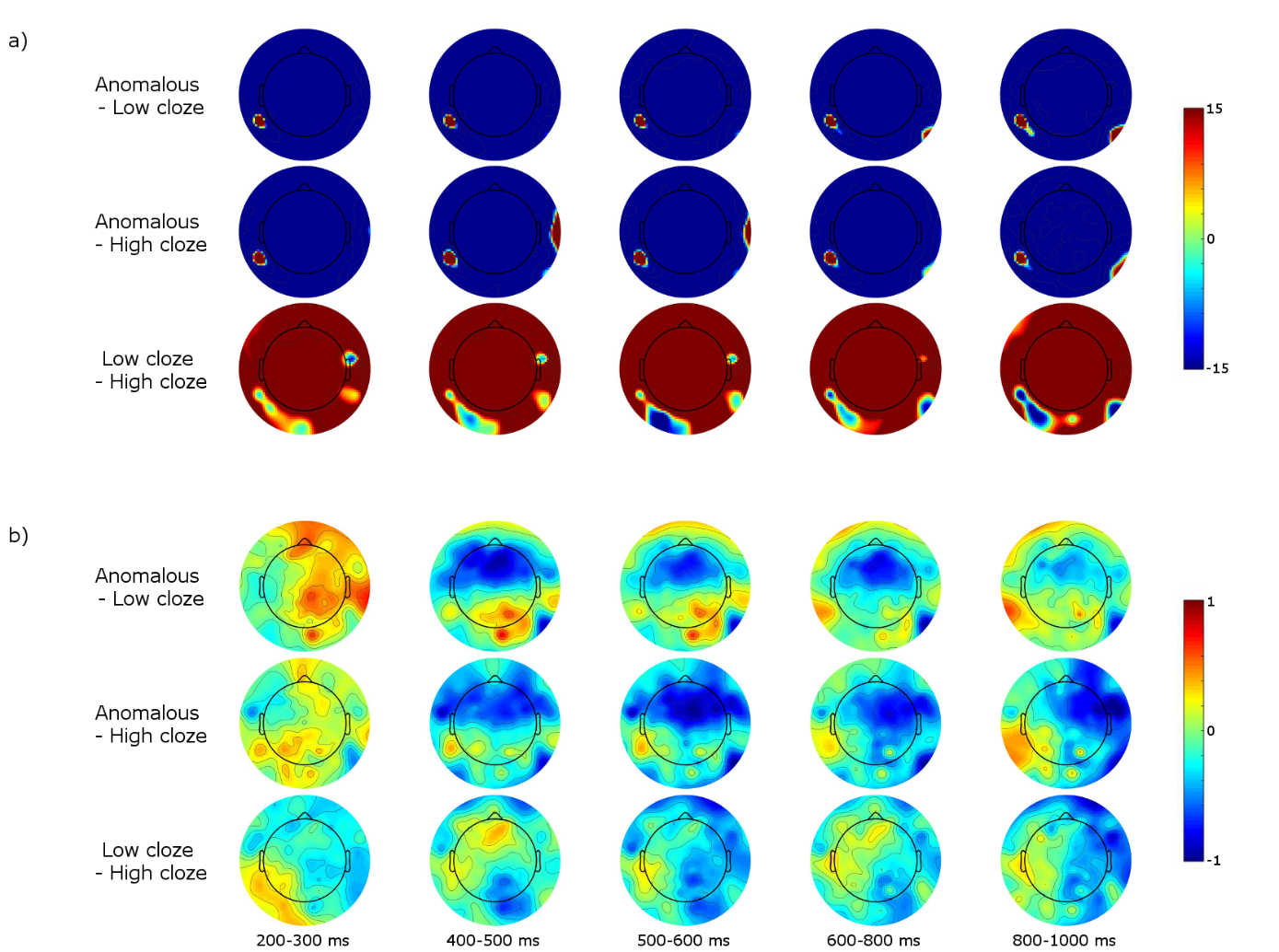


Supplementary Figure S2: Scatterplot of correlations between cloze rating (including every item) and amplitude at the C4 (left) and P4 (right) clusters from 500-600 ms. Negative is plotted upwards.


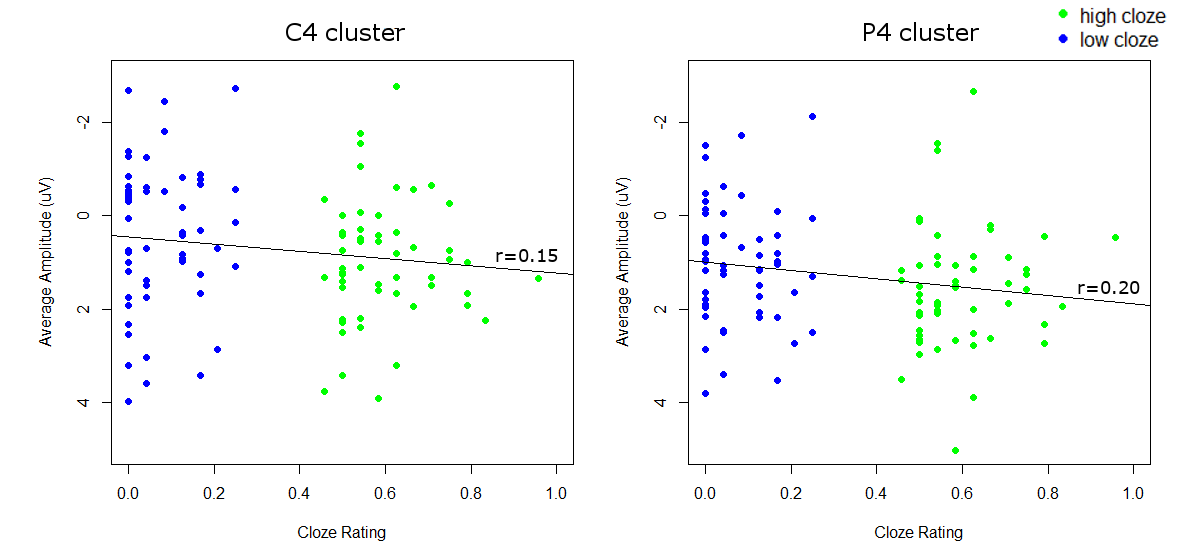


Supplementary Figure S3: Mean ERP waveforms at the critical panel for each condition at nine cluster across the scalp. Shaded regions for each condition indicate the standard error at each timepoint. Negativity is plotted upwards.


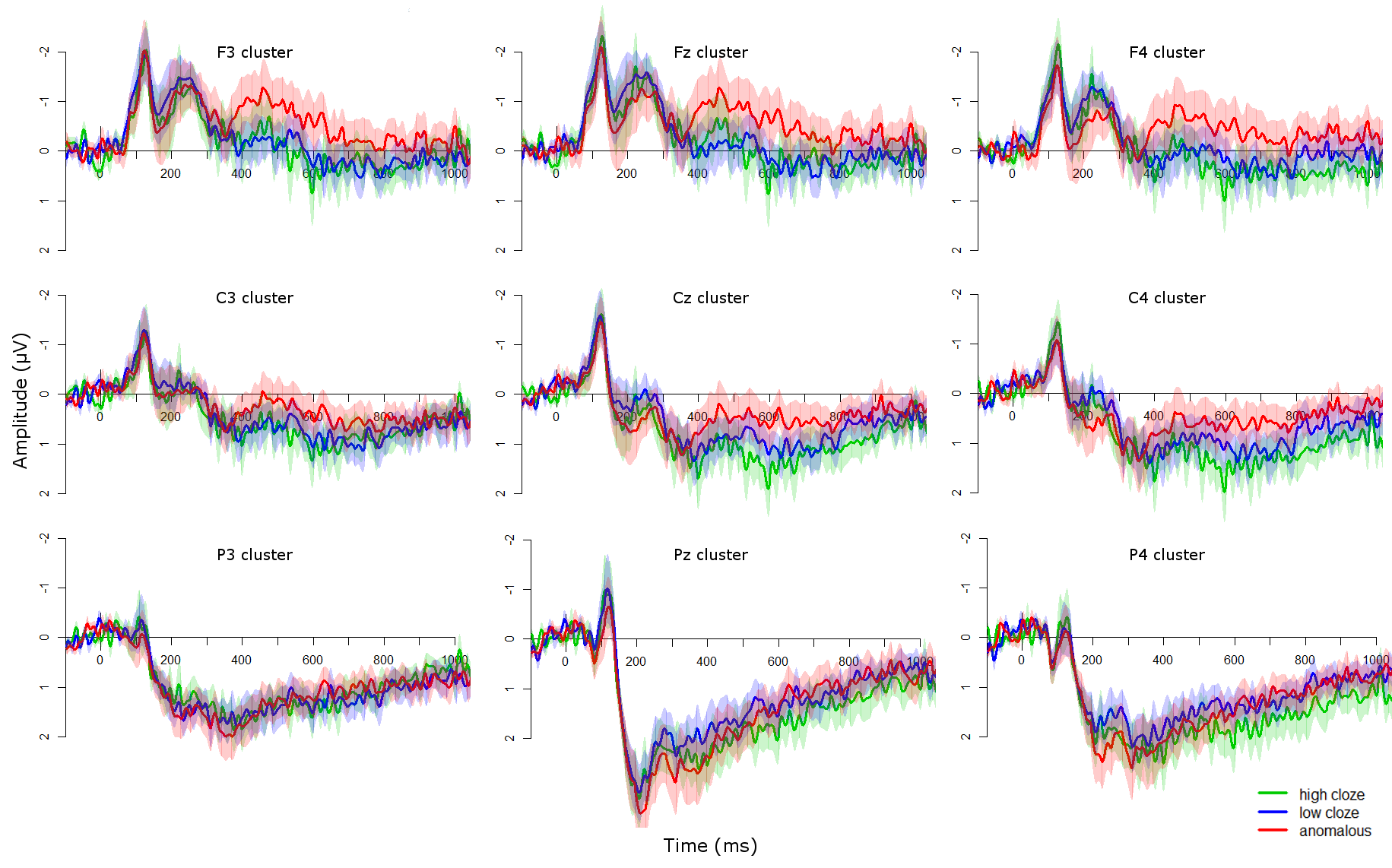


Supplementary Figure S4: Boxplots of mean ERP waveforms at the critical panel from 500-600 ms for each condition at nine cluster across the scalp. Negativity is plotted downwards
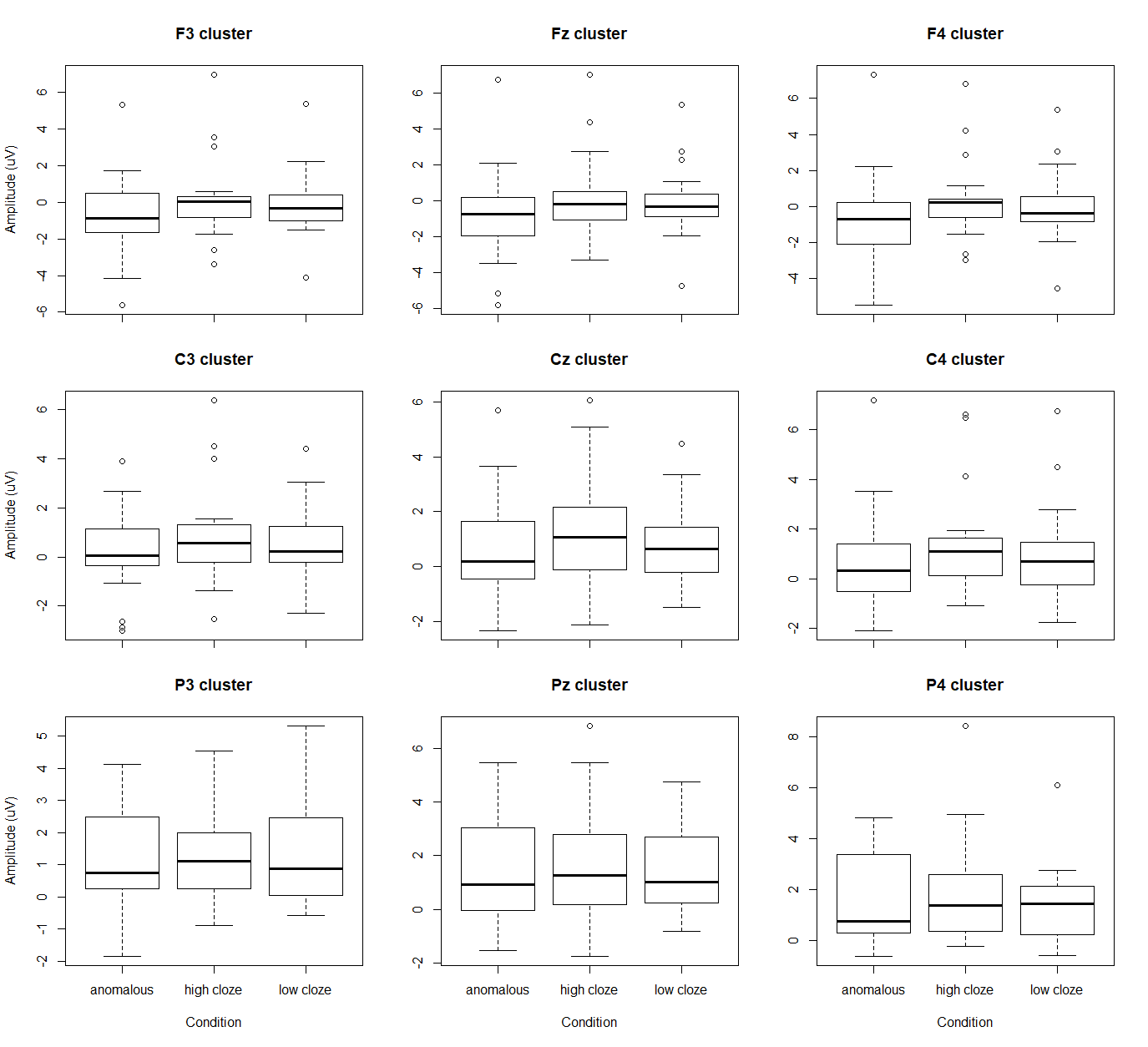
.

Supplementary Table S1: Examples of responses from the cloze ratings for the high-cloze example strip shown in Figure 1.

| **Answer** | **Exact match** |
| --- | --- |
| He is knocked over by the boxing figure. | 1 |
| He is knocked down by the toy. | 1 |
| The bouncy toy will hit the boy back. | 1 |
| The bopper hits the kid. | 1 |
| The boy is knocked over by the balloon robber. | 1 |
| He accidentally punches someone. | 0 |
| The thing bounces back, knocking him over. | 1 |
| The punching doll knocks him down. | 1 |
| The punching bag knocks him over. | 1 |
| The punching bag rocks back and hits the kid. | 1 |
| It bounces back and knocks him over. | 1 |
| The toy knocks him over. | 1 |
| The toy hits the boy, who cries. | 1 |
| The inflatable punching bag pops back up right into his face and knocks him to the ground. | 1 |
| The bouncy fighter knocks the boy out. | 1 |
| The punching bag comes back to hit him. | 1 |
| Bop-bag comes back up and hits him. | 1 |
| Bozo blow up hits the boy to the ground. | 1 |

Supplementary Table S2: Number of trials included for each participant and condition (out of max of 60)

| **Participant** | **High cloze** | **Low cloze** | **Anomalous** |
| --- | --- | --- | --- |
| 1 | 53 | 51 | 49 |
| 2 | 48 | 48 | 48 |
| 3 | 50 | 52 | 54 |
| 4 | 42 | 52 | 42 |
| 5 | 40 | 39 | 43 |
| 6 | 45 | 43 | 45 |
| 7 | 48 | 54 | 50 |
| 8 | 45 | 47 | 53 |
| 9 | 57 | 54 | 57 |
| 10 | 39 | 47 | 49 |
| 11 | 32 | 29 | 33 |
| 12 | 51 | 48 | 49 |
| 13 | 28 | 27 | 31 |
| 14 | 47 | 50 | 51 |
| 15 | 52 | 51 | 45 |
| 16 | 39 | 48 | 52 |
| 17 | 48 | 53 | 49 |
| 18 | 40 | 32 | 41 |
| 19 | 38 | 38 | 36 |
| 20 | 40 | 40 | 34 |
| 21 | 42 | 42 | 45 |
| 22 | 30 | 31 | 35 |
| **average** | **43** | **44** | **45** |
| **sum** | **954** | **976** | **991** |
